# Future climate response to observed strong El Niño analogues

**DOI:** 10.1038/s41612-025-01003-1

**Published:** 2025-03-21

**Authors:** Paloma Trascasa-Castro, Yohan Ruprich-Robert, Amanda C. Maycock

**Affiliations:** 1 Earth Sciences Department, Barcelona Supercomputing Center, Barcelona, Spain; 2 School of Earth and Environment, University of Leeds, Leeds, UK

**Keywords:** Ocean sciences, Climate change, Climate-change impacts, Climate sciences, Atmospheric science, Atmospheric dynamics

## Abstract

The effect of future climate change on the boreal winter response to strong El Niño is investigated using pacemaker simulations with the EC-Earth3-CC model constrained towards observed tropical Pacific sea surface temperature anomalies. Under the Shared Socioeconomic Pathway 2-4.5, the surface temperature response to strong El Niño intensifies in North America, northern Africa, Australia and the North Atlantic compared to present day. However, future strong El Niño has a weaker climate impact in southern America and Africa. Temperature extremes under strong El Niño intensify in the future in some regions, with more cool days in eastern North America, while warm days in northern South America decrease. Assuming that the characteristics of strong El Niño events will not change in the future, we distinguish between changes in El Niño teleconnections and background climate changes, and found that the latter dominates the absolute climate response to strong El Niño events.

## Introduction

El Niño-Southern Oscillation (ENSO) is the dominant mode of interannual climate variability across the global tropics. Every 2-7 years, anomalously warm or cool sea surface temperature (SST) anomalies develop in the equatorial Pacific Ocean and trigger a global cascade of remote effects^1^. The warm phase of ENSO, known as El Niño, leads to dry and hot conditions over Australia^2^, northern South America^3^, southern Africa^4,5^ and the Indian Summer monsoon region^6^, as well as wet and cooler conditions over some parts of North America^7^ and Eastern Africa^8^.

The strongest El Niño events since the late 19^th^ century occurred in 1982/83, 1997/98, and 2015/16, when SST anomalies in the Niño3.4 region (SST averaged over 5°S-5°N, 170 W°-120°W) reached 2.2^o^C, 2.4^o^C and 2.6^o^C, respectively (estimated as 3-month running means with the ERSST.v5 dataset^9^). These strong El Niño events led to widespread devastating impacts, including severe droughts in Southeast Asia, Northern and Southern Africa^10^, the Amazon^11^, Mexico and Philippines, and a mangrove dieback in Northern Australia^12^; widespread fires in Indonesia^13,14^; storms in the US, flooding in Cuba, Peru, Bolivia and Ecuador^15^; large changes in the global carbon cycle^16,17^; and food insecurity^10,18^.

During strong El Niño events, SST and precipitation anomalies extend further east in the equatorial Pacific compared to moderate events^19–21^ particularly in December-January-February (DJF). The teleconnections of El Niño to the Indo-Pacific sector^22^, Australian^23^, Northern^24^ and South American^25^ and North Atlantic-European^26^ regions depend on the amplitude of the event, with the impacts of strong El Niño often more severe than weak or moderate events.

The global impacts of strong El Niño events may change in the future due to increases in anthropogenic radiative forcing and, as a direct consequence, continued global mean surface warming. Such changes could arise from two effects: 1) changes in the frequency and/or characteristics of strong El Niño events; 2) changes in the processes that lead to teleconnections. Both effects depend on the interactions between ENSO and the mean climate state. Regarding mechanism 1, in the tropical Pacific most climate models project a mean weakening of the Walker circulation and a reduction of the zonal SST gradient in the equatorial Pacific by the end of the 21^st^ century^27^. This change in mean state leads to an increase of ENSO variability under increased anthropogenic forcing in most models from the Sixth phase of the Coupled Model Intercomparison Project (CMIP6)^28,29^, including an increase in the likelihood of strong El Niño events^20,30^ partially driven by a strong increase in temperature over the central and eastern equatorial Pacific. However, there is uncertainty associated with this response, in part due to the generally poor representation of recent tropical Pacific SST trends in historical model simulations^31^. In fact, despite most CMIP6 models simulating an “El Niño-like” SST state in the future, the models which more closely capture the observed the tropical Pacific SST trend project a continuation of the “La Niña-like mean state”, which eventually might weaker the amplitude of El Niño events in the future^32^.

Regarding mechanism 2, the Sixth Assessment Report of the Intergovernmental Panel on Climate Change highlights a robust trend towards an increase in ENSO-driven precipitation variability in a warmer climate^33^. Following the Clausius-Clapeyron relationship, absolute humidity increases by approximately 7% per Kelvin, hence the changes in tropical humidity driven by ENSO are expected to increase in the future^34^. Ying et al.^35^ found that an anthropogenically-forced signature in ENSO-related precipitation variability will emerge around 2040, decades before a robust change in ENSO SST can be detected in models^27^. This means that we might expect changes in ENSO teleconnections to emerge before any changes in ENSO characteristics may be detectable from internal climate variability.

While several modelling studies have examined ENSO teleconnections in a future climate, they have generally used freely-evolving coupled climate models and therefore do not permit a separation of mechanisms 1 and 2^29,36–39^. McGregor et al.^38^ found significant changes in ENSO impacts over around one third of global land area in CMIP6 models, but it was unclear what factors caused these changes. A small number of studies have isolated mechanism 2 by prescribing identical SST anomalies on top of present and future background climates^40,41^. These studies found an amplification of ENSO teleconnections in boreal winter in the North Pacific and North Atlantic-European sectors under future climate conditions. However, Drouard and Cassou^40^ used ENSO SST anomalies derived from a model, which may not be fully realistic, and Zhou et al.^41^ used an atmospheric model meaning any role of atmosphere-ocean coupling in modifying teleconnections was excluded. Neither study assessed the global effects of future climate changes on El Niño impacts and did not specifically address the impacts of strong El Niño events, which as noted above are often the most impactful.

Our study follows a similar approach to Drouard and Cassou^40^ and Zhou et al.^41^ by exploring mechanism 2 and assuming that ENSO characteristics do not vary with climate change. We specifically focus on strong El Niño and use observed SST anomalies combined with a pacemaker modelling approach to enable atmosphere-ocean coupled interactions outside of the tropical Pacific (see Methods). We simulate three observed strong El Niño events (1982/83, 1997/98 and 2015/16) under present day and projected future climate states corresponding to the year 2090 under the SSP2-4.5 scenario. We focus on responses in the boreal winter season (DJF) and find the events behave similarly in our pacemaker simulations, so in most of the analyses we combine them into a multi-event mean (MEM).

## Results

### Changes in the tropical Pacific induced by strong El Niño and climate change

**Fig. 1:**
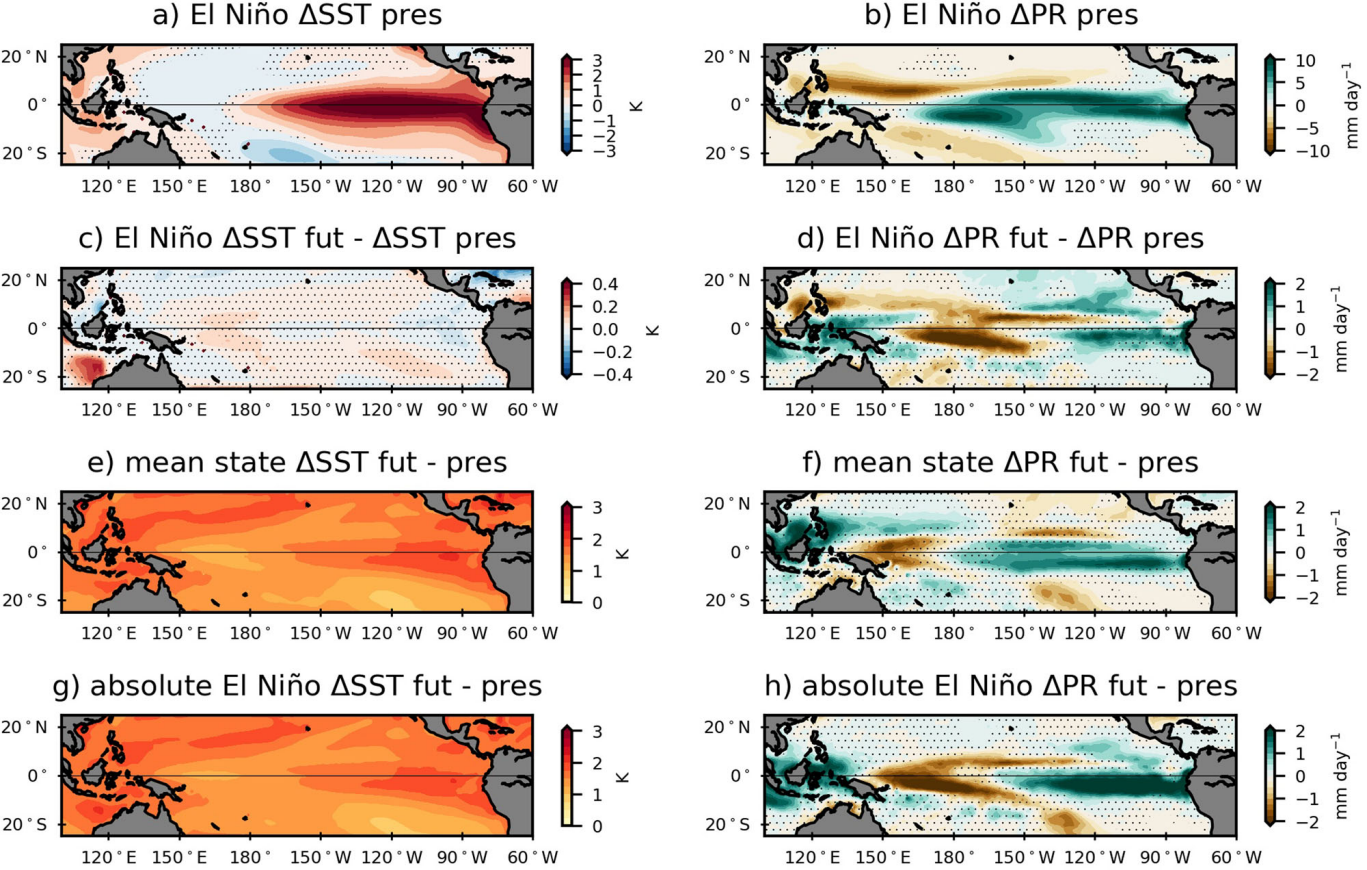
Changes in the tropical Pacific induced by strong El Niño and climate change. DJF SST (K, left) and precipitation (mm day^−1^, right) anomalies during El Niño in the present (**a**, **b**), differences between future and present El Niño anomalies (**c**, **d**), the mean state change (**e**, **f**) and difference in El Niño states between future and present day (**g**, **h**). Hatched areas are not statistically significant at the 95% confidence level.

Figure 1a shows the MEM El Niño SST anomalies under present day. The anomalies closely resemble the pacemaker target SST pattern, with the canonical eastern Pacific structure of strong El Niño. In the tropical Pacific, strong El Niño events drive an eastward shift of the precipitation maximum, leading to precipitation anomalies of above 10 mm day^−1^ over the central and eastern equatorial Pacific (Fig. 1b), similar to the observed precipitation pattern during El Niño (Fig. S1). Those positive anomalies contrast with drier conditions off the equator around 10°N and in the South Pacific Convergence Zone.

There are very small differences in the El Niño SST anomalies in the future (Fig. 1c), indicating the pacemaker method works as intended (see “Methods” and Fig. S2). There are, however, significant differences in El Niño-driven precipitation in the future (Fig. 1d), with higher precipitation rates in the eastern (more wetting) and western (less drying) equatorial Pacific of up to 2 mm day^−1^ and negative anomalies (less wetting) of at least 2 mm day^−1^ over the central equatorial Pacific. We note that these differences do not fully match the projected change in El Niño-driven precipitation anomalies in the tropical Pacific suggested by the literature, consisting of an eastward shift of precipitation during El Niño under greenhouse^38^ forcing^42^. This might indicate an important role for El Niño-SST changes in the future El Niño-precipitation relationship seen in coupled climate models, or be specific to the effect of climate change on the precipitation response to strong El Niño events, which are generally not isolated in other studies. It is also possible the pacemaker set–up influences the associated precipitation response, for example because it constrains surface turbulent fluxes, but similar differences were found in an experiment with weaker flux restoring (not shown).

In terms of mean state changes, the tropical Pacific warms by around 2.5 K in the future (Fig. 1e), with the Niño4 (5°N-5°S, 160°E-150W) and Niño3 (5°N-5°S, 150°W-90°W) regions being 4.7% and 6.6% warmer, respectively, compared with the present day. Consequently, the zonal east-west equatorial Pacific SST gradient decreases by 19.5%, similar to the response in other CMIP6 models^43^. Coincident with the mean SST changes, there is a weakening of the Walker circulation, evidenced by a decrease in precipitation over the warm pool and an increase in precipitation over the Central and Eastern Pacific (Fig. 1f).

To understand how strong El Niño conditions would appear in the future compared to present day, we next consider the combined mean state plus El Niño anomalies (Fig. 1g and 1h). Since the El Niño SSTs are largely unchanged in the future, the total near-surface temperature signal mainly reflects the change in tropical Pacific mean state, whereas for the total precipitation anomaly, the substantial negative anomalies between 170°E-150°W seen as a difference in the response to El Niño in the future, expand the location of dry conditions further towards the central Pacific. Such a change might be expected to alter the teleconnections from El Niño, which is explored in the next section.

### Teleconnections from strong El Niño events are modulated by climate change

**Fig. 2:**
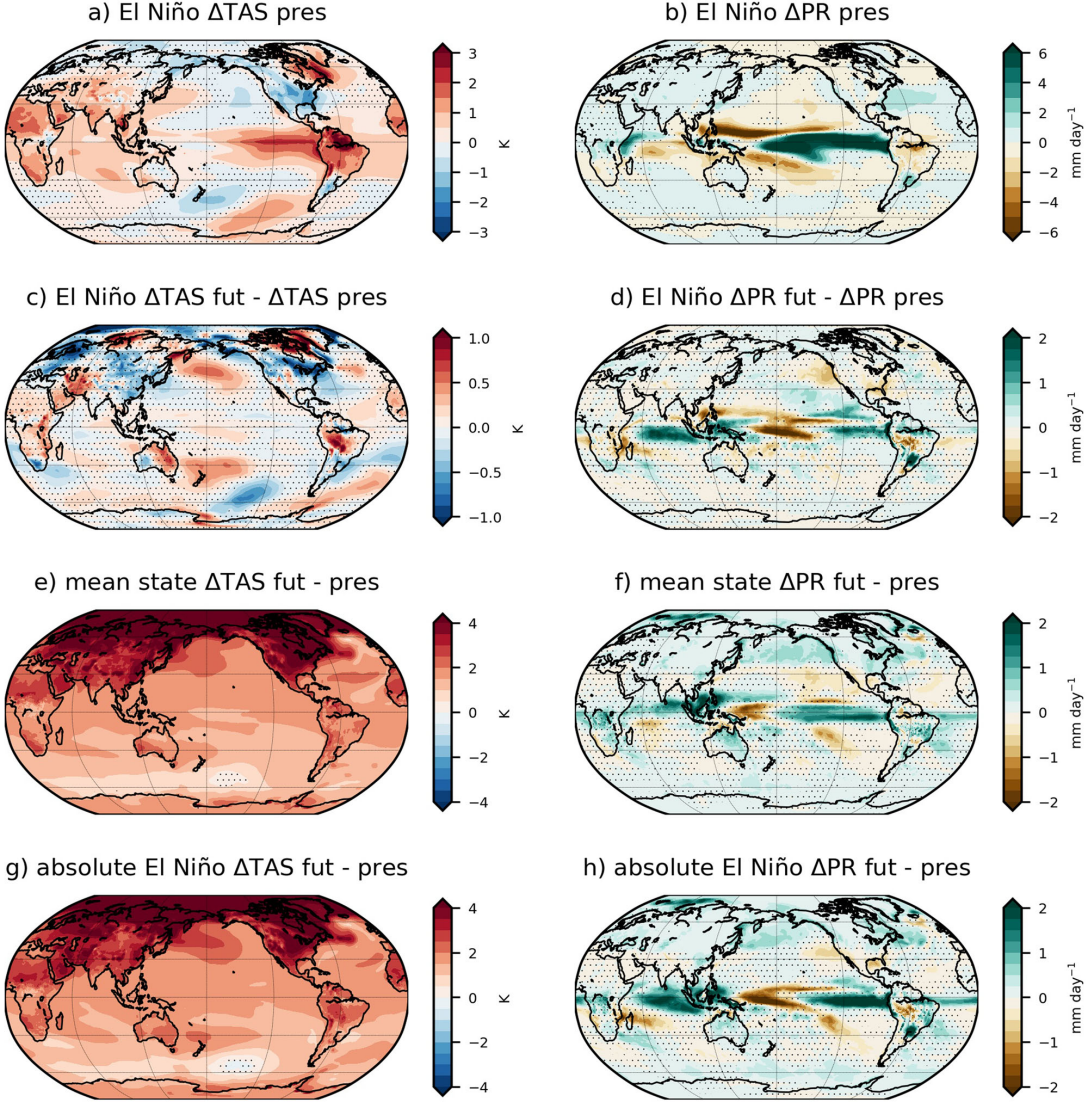
Future global changes in strong El Niño teleconnections in DJF. Surface temperature (K, left) and precipitation (mm day^−1^, right) anomalies during El Niño in the present (**a**, **b**), differences in future and present El Niño anomalies (**c**, **d**), mean state changes (**e**, **f**) and changes in absolute values between future and present (**g**, **h**). Hatched areas are not statistically significant at the 95% confidence level.

In the present day, the modelled strong El Niño drives significant surface temperature impacts across the globe in DJF (Fig. 2a). Northern South America experiences warm anomalies reaching up to 3 K over the Amazon, as well as large parts of the African continent, with the exception of near Lake Victoria. Western Australia experiences hotter summers during strong El Niño, and south Asia also sees warm anomalies. There are some regions, though, where El Niño leads to cold anomalies in DJF, such as the southern and western US and south-east South America (SESA). Note that the EC-Earth3-CC model captures the amplitude and spatial location of the winter surface response to El Niño over northern South America, North America and Southern Africa similar to observations (Fig. S1).

Alongside surface temperature changes, strong El Niño events drive dry anomalies of up to 2 mm day^−1^ over the Amazon basin, although based on the model evaluation these anomalies may be overestimated (Fig. S1), and around 1 mm day^−1^ over Southern Africa. There is a dipole precipitation anomaly over the North Atlantic, likely driven by a southward shift of the North Atlantic jet stream and storm track (Fig. 2b), characteristic of the link between El Niño and the negative phase of the winter North Atlantic Oscillation (NAO)^44^. Over the Congo basin and the Horn of Africa, strong El Niño leads to weak but significant positive precipitation anomalies. Precipitation also increases by 2 mm day^−1^ over SESA during strong El Niño, leading to wetter than usual summers in the north of Argentina, Uruguay and Paraguay. EC-Earth3-CC effectively captures the sign and spatial location of positive precipitation anomalies during El Niño, but slightly underestimates its amplitude compared to observations (Fig. S1). In terms of the ability of the model to represent the complexity of the amazon climate, it has shown to correctly simulate the annual cycle of temperature and precipitation, and outperforms most CMIP6 models in the representation of the south American monsoon dynamics^45^.

In the future, the DJF surface temperature response to strong El Niño increases in several regions (Fig. 2c). Strong El Niño drives even hotter conditions in most of Africa (except in its southern part), and hotter summers in the south-west of the Amazon and Congo basins and eastern Australia, being the future response to El Niño in the latter stronger than in the present. Over north-east North America, the cold anomalies driven by strong El Niño are also enhanced in the future. On the other hand, mean DJF temperature responses driven by strong El Niño in the future weaken in other regions potentially due to shifts in atmospheric circulation. For example, cold anomalies over north-east Argentina, Paraguay and south Brazil extend more towards the north, following the northward shift of precipitation shown in Fig. 2d. Over southern Africa, the temperature and precipitation responses to strong El Niño weaken in our simulation, leading to milder summers.

In Australia, the influence of future climate change on the response to strong El Niño shows dipole anomalies consisting of higher precipitation rates over western Australia and drier conditions over the east in DJF, which is opposite to the projected precipitation trend in austral summer in the model (Fig. 2f). We note that other climate models tend to show an increase in Australian summer rainfall in future projections^33^, which would constructively interfere with the altered strong El Niño signal in western Australia. In a limited number of regions, the precipitation response to strong El Niño events weakens in the future compared to present day. For instance, in the present day strong El Niño drives dry anomalies over the Amazon basin (Fig. 2b), but this changes in the future, where the meteorological drought induced by strong El Niño becomes significantly weaker (Fig. 2d).

Figure 2e, f show the mean state surface temperature and precipitation changes between the future and present day. By the late 21^st^ century, EC-Earth3-CC simulates an overall statistically significant increase in temperature across the globe, especially in the Northern Hemisphere (in agreement with Lee et al.^33^), where land areas experience a warming of above 4 K in DJF. In the Southern Hemisphere, regions like western Australia, southern Africa and the Amazon basin are warming faster than others. In terms of precipitation, besides the projected eastward shift in tropical Pacific precipitation resulting from a future weakening of the Walker circulation, there’s an overall increase in precipitation across the tropics, in agreement with CMIP6 projections^33^. Regions like Australia, North America and East Asia are expected to experience a slight increase precipitation in DJF, in agreement with multimodel projections, although the change in precipitation is not as evident as in temperature due to the higher variability of the former and the larger model uncertainty regarding dynamical changes as a response to climate change. The total anomalies during strong El Niño in the future compared to present day (Fig. 2g, h) show constructive interference and exacerbated El Niño-driven warm anomalies over eastern Australia, Northern South America and Central Africa during the austral summer months, which would lead to increased heat-related risks^46,47^. The mean warming under climate change masks any enhancement of the cooling effect of strong El Niño, such as over North America or East Asia. Nevertheless, over South America, Fig. 2h highlights the contribution of changes in strong El Niño impacts to the absolute response over the Amazon basin, which drives an increase in precipitation in DJF, opposing the mean drying under global warming.

### Tropospheric circulation response to strong El Niño and its changes under future conditions

**Fig. 3:**
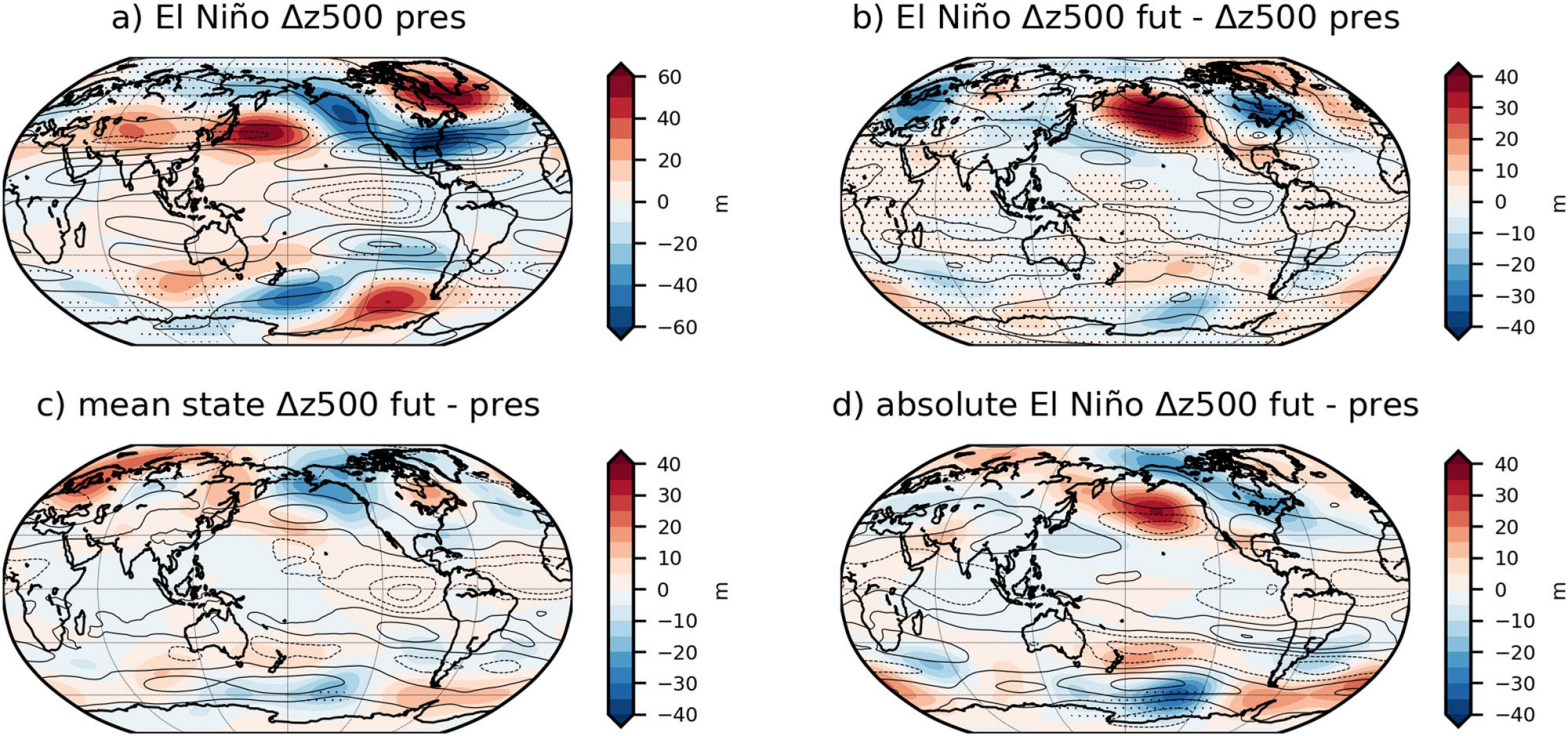
Future changes in mid-tropospheric circulation due to strong El Niño and climate change. Eddy geopotential height at 500 hPa, (deviations from the zonal mean, m, filled contours) and zonal wind at 200 hPa anomalies (m s^−1^, black contours, where solid lines denote positive anomalies and dashed lines show negative anomalies) during El Niño in the present (**a**), differences in future and present El Niño anomalies (**b**), mean state changes (**c**) and changes in absolute values between future and present (**d**). Hatched areas are not statistically significant at the 95% confidence level.

To understand future changes in teleconnections of strong El Niño, we explore tropospheric geopotential height at 500 hPa (z500) and zonal wind at 200 hPa (u200) anomalies in DJF. The extratropical teleconnections of El Niño depend on the background flow, specifically on the upper tropospheric zonal wind^48^, which is stronger in the Northern hemisphere in DJF due to a stronger subtropical jet. In the present day, strong El Niño generates positive z500 anomalies of up to 60 m off the coast of Japan (Fig. 3a), a deepening of the Aleutian low and barotropic Rossby wave trains that project onto the Pacific South American (PSA1) and Pacific North American (PNA) patterns. As opposed to moderate and weak El Niño events, strong episodes shift the PNA further east^24^, making these events unique and incomparable with El Niño events of lesser amplitude.

Outside of the Pacific sector, strong El Niño drives negative geopotential anomalies over the south-east USA, which extend eastward over the subtropical Atlantic Ocean. Those negative anomalies contrast with positive anomalies spanning from the north-east of Canada to the Norwegian Sea. This corresponds to a negative phase of the NAO and a southward shift of the North Atlantic westerly jet stream, in agreement with the southward shift of precipitation anomalies shown in Fig. 2b.

Under future conditions, strong El Niño drives a weaker PNA response, with the simulations showing a relatively stronger Aleutian low in boreal winter with positive z500 anomalies of above 40 m (Fig. 3b). The eastward shift of z500 anomalies in the North Pacific and North America, in agreement with Johnson et al.^49^ and Geng et al.^50^ coincide with below normal temperatures shown in Fig. 2c. Similar conditions are shown over central and northern Europe, where strong El Niño events drive relatively low pressure anomalies associated with colder winters. There is a high pressure anomaly in the Caribbean region during strong El Niño events in the future, which translates into the drying trend shown in Fig. 2c. In the Southern hemisphere, strong El Niño triggers an anomalous wavetrain spanning the extratropics and subpolar regions and driving a dipole of positive and negative z500 anomalies in the west and eastern coasts of South America, respectively. The projected changes in El Niño-driven z500 over South America, do not resemble those shown in Johnson et al.^49^ possibly due to the weaker climate change signal in our simulations (compared to SSP5-8.5 used in their study) and the fact that changes in El Niño anomalies modulated by climate change are only expected to emerge at the end of the century. Also, as it is the case of the teleconnection towards north America, the fact that amplitude of the regional response to El Niño is not linearly related to the strength of the event makes it hard to compare with studies based on El Niño composites of all strengths as in Johnson et al.^49^ and McGregor et al.^38^.

In terms of mean state changes, Fig. 3c shows a deepening of the Aleutian low by 20 m in the future climatology, compared to present-day conditions. In the South Pacific Ocean, there is a strengthening of the zonal low pressure anomalies across SESA, and positive z500 anomalies in the Southern Ocean off the coast of South America. It is important to note that the amplitude of future changes in El Niño-driven z500 anomalies (Fig. 3b) is twice as large as the mean state changes (Fig. 3c), highlighting the significance of changes in ENSO teleconnections in the future relative to changes in the background flow. The absolute changes in z500 and zonal wind during strong El Niño events in the future are dominated by changes in El Niño teleconnections rather than by mean state changes, as suggested by Beverley et al.^51^.

### Regional extreme temperature responses to strong future El Niño events

Building on the evidence in the previous sections showing regional signatures of the modulation of strong El Niño teleconnections under future climate change, we now investigate regional changes in meteorological extremes under strong El Niño, in North America, southern South America and southern Africa. Special attention is paid to shifts in the distribution of daily maximum temperature from the present to the future considering both cool and warm days (see Methods).

We first consider cool days in North America using a cool day index (see Methods) to understand changes to the distribution of cold anomalies driven by El Niño in the future. Note this index is percentile-based and therefore effectively removes the climatological shift in cool days driven by anthropogenic forcing.

**Fig. 4:**
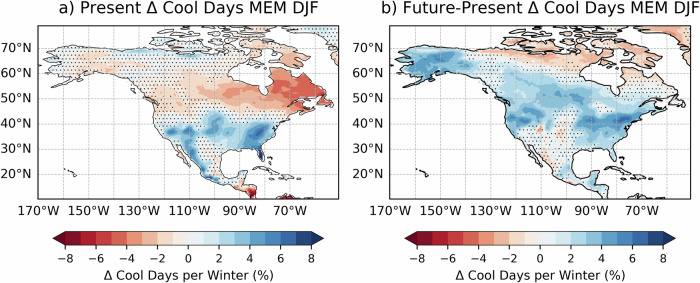
El Niño-driven cool days in North America. Anomalous cool days per winter (DJF) during strong El Niño in North America in (**a**) present conditions and (**b**) future - present conditions. Only grid cells corresponding to land surface areas are used in this plot. Areas where strong El Niño events do not drive statistically significant anomalies in the present-day simulations appear as hatched in subpanel (**a**). Hatching in subpanel (**b**) show non significant changes in the impacts of strong El Niño events between the future and present-day simulations.

In the present day, strong El Niño drives an increase of cool days in North America of up to 7% per winter, especially along the Rocky Mountains and central-eastern US (Fig. 4a), and a 6% reduction in cool days per winter over eastern Canada. There is a relative increase in cool days per winter during strong El Niño events over most of the USA in the future compared to present day (Fig. 4b), meaning an intensification of cold anomalies in the east and the appearance of cool anomalies over the northern US and Alaska. The mean climate change signal shifts the local climatological 10^th^ percentile of maximum daily temperature towards higher values in the whole North America (See Fig. S3c), especially over the Northeast of the United States, Canada and Alaska, where the threshold for cool days increases by up to 8 °C. The changes in relative occurrence of cold days during strong El Niño could have societal implications, since a focus on adaptation to a warmer climate may reduce the focus on preparedness for cold days. At the same time, percentile-based indicators might not be as good a proxy for climate impacts on health in the future as they are in the present, i.e. cold days in the future will be less harmful for human health. This difference overrides the intensification of the cold El Niño anomalies expected at the end of the century, since the overall temperature will be higher due to the dominance of the global warming signal for the total temperature anomaly.

Northern South America is one of the regions where strong El Niño events have the largest impacts on daily temperature extremes. From December to February, strong El Niño leads to an increase of warm days of up to 40%, especially around the Amazon basin in Brazil, Venezuela and the Pacific coast of Colombia and Ecuador. In the future, the striking impact of strong El Niño events in the region gets muted in the Amazon basin, where the percentage of warm days decreases up to 15%. In southwest Brazil and Bolivia, however, there is a significant strengthening of the El Niño signal, with up to 15% more warm days from December to February. Changes in extreme temperature impacts driven by El Niño in the future will play a key role in the frequency and intensity of forest fires in the Amazon region, fueled by a negative precipitation and soil moisture trend^52^.

According to Fig. 6, during strong El Niño, areas of Africa south of the equator experience a strong increase in warm days in summer, with the largest increases over Equatorial Guinea, Republic of the Congo and Angola, with up to 25% additional warm days per summer. In the future experiment, there is a relatively smaller increase in warm days per summer due to strong El Niño (approximately 7% less in some areas). In addition, there are enhanced warm anomalies towards the northern regions of lakes Tanganyika and Malawi, Tanzania and the Democratic Republic of the Congo, where the number of anomalous warm days per summer during strong El Niño is found to increase by up to 10%.

Figure S3g-i shows the shift of the 90^th^ Tmax percentile towards higher values in southern Africa, especially over the west and southwest of the region, where the temperature threshold for a day to be considered warm will rise from 30 °C to 35 °C. The smaller increase in warm days in the future relative to present-day conditions is caused by a raise of the Tmax threshold for warm days. Nevertheless, in the future strong El Niño still leads to an increase in warm days in southern Africa, further exacerbating the underlying signal of climate change consisting of a shift towards higher temperatures in the region in austral summer. In the coastal areas of Mozambique and Tanzania there is a significant increase of up to 10% in the number of El Niño-driven warm days in summer, which constructively interfere with climate change leading to an exacerbation of warm summer days in the region. As shown in Figure S3i, the largest shift in the climatological warm day threshold is found in Namibia, Botswana and Mozambique.

## Discussion

This study has used idealised pacemaker simulations with the EC-Earth3-CC model following the SSP2-4.5 scenario to quantify changes in the global impacts of strong El Niño events in a warmer climate. This approach isolates the role of changing background climate conditions on the atmospheric response to El Niño, which has been a limitation of some previous studies (e.g., McGregor et al.^38^).

The experiments show precipitation anomalies during strong El Niño increase in the eastern equatorial Pacific and warm pool under future climate change, and decrease over the central Pacific (Fig. 1d). The changes in the strong El Niño precipitation response in the eastern Pacific are comparable to the changes from mean climate change. Alongside the impact of climate change on the local Pacific response to strong El Niño, the large-scale global atmospheric teleconnections are also modified. The Rossby wave trains emanating from the western tropical Pacific into the northern and southern hemispheres show differences in amplitude and phase that modify regional climate hazards associated with strong El Niño.

The change in frequency of cool days per winter under strong El Niño is larger in the future over eastern North America. It is important to note that due to the overall background warming from climate change, the percentile-based threshold adopted for cool days increases from −5 °C to 0 °C between present and future meaning the absolute temperatures are less severe but still below freezing. The number of cool days per winter slightly decreases in the future climatology. Owing to the altered teleconnection associated with the PNA pattern, there is a larger frequency of cool days driven by El Niño in the future (17.1%) than in the present (9.5%), as suggested by Lieber et al. ^5^. In agreement with Meehl et al.^53^ we find an eastward shift of cold extremes in future El Niño events and a decrease in cool days over the west coast of the USA. This shift might be linked to the strengthening of the teleconnection of El Niño to the North Atlantic sector^54^.

**Fig. 5:**
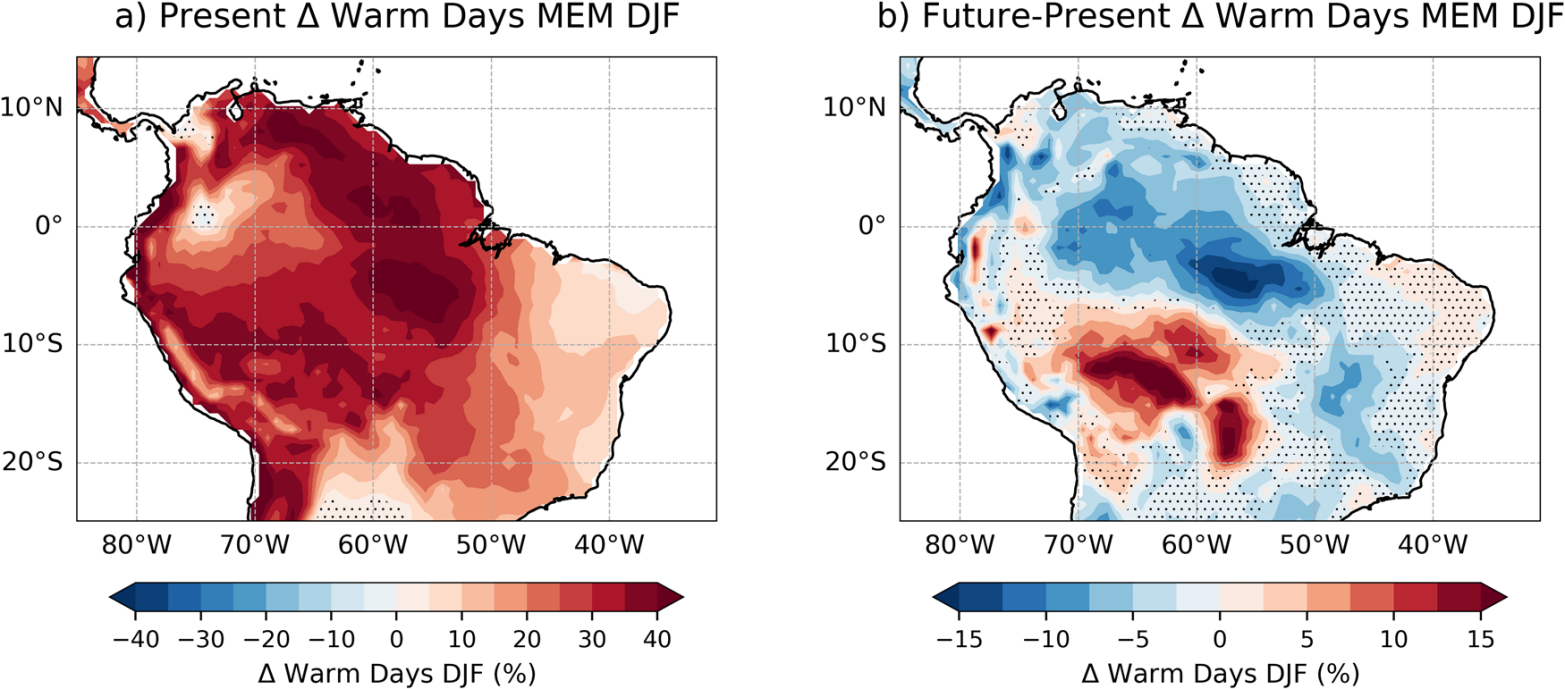
El Niño-driven warm days in Northern South America. Anomalous warm days per summer (DJF) during strong El Niño in Northern South America in (**a**) present conditions and (**b**) the difference between future and present conditions. The hatching convention is the same as in Fig. 4.

Finally, we show that despite most of the regions with significant changes in strong El Niño impacts, a limited number of areas, like northern South America and southern Africa, experience a weakening of the signal driven by El Niño under future conditions. In the Amazon basin, a region where warm days roughly double in frequency when there is a strong El Niño event, our experiments suggests a weakening of this teleconnection translated into a decrease in warm days over most of the region. Figure 5 shows a weakening of the El Niño-driven warm days in most of the region except southeast Brazil and Bolivia, which might be the result of a saturation of the impacts of El Niño under events of high amplitude such as the ones considered in this study. The weakening of El Niño-driven temperature extremes in northern South America in the future coincides with the expected changes in mean temperature (Fig. 2c).

**Fig. 6:**
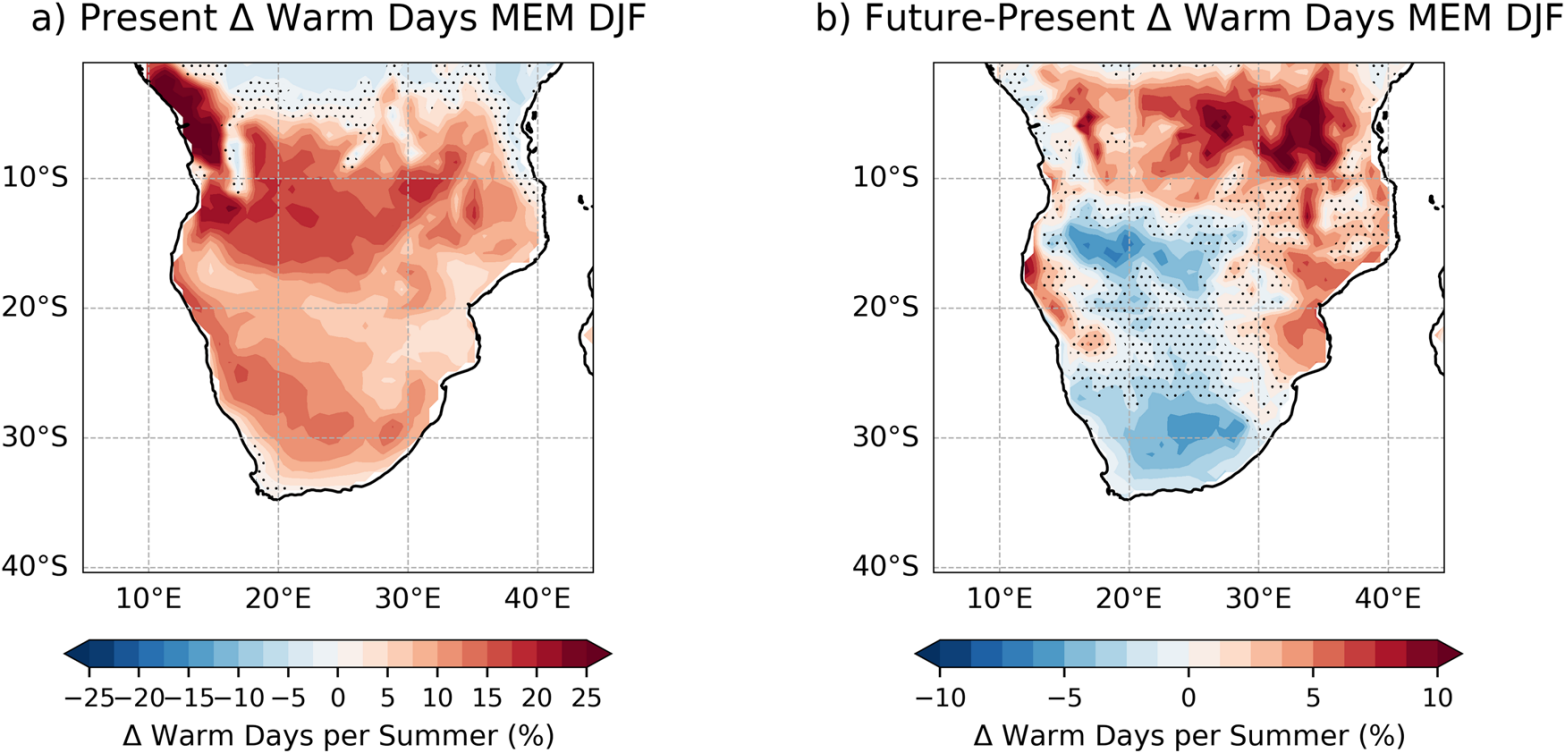
As in Fig. 5, but for Southern Africa.

Figure 6 suggests that El Niño-driven warm days in summer will be lower in the future, especially over southern Namibia, Botswana and central South Africa. While the threshold to compute warm days as simulated by EC-Earth3-CC increases from 28.8 °C in the present to 31.2 °C in the future, in agreement with future CORDEX-Africa climate projections over the region^55^, the El Niño-driven warm days do not increase in frequency in a warmer climate. In absolute terms, the background warming induced by climate change will increase the frequency of heatwaves over Southern Africa, independent of whether there is a strong El Niño event happening or not.

Interestingly, the projected changes in El Niño teleconnections resemble those identified in previous studies, suggesting that simulated changes in future impacts of El Niño in coupled climate models are dominated by changes in the mean state, rather than by changes in ENSO SSTs. In agreement with McGregor et al.^38^, we find a strengthening and southward shift of the North Atlantic jet stream in boreal winter during El Niño events in the future, highlighting the reinforcement of the El Niño-negative NAO relationship. This is also in agreement with previous literature (i.e. Drouard and Cassou^40^, Johnson et al.^49^), although this response is not captured in CMIP5 models^37^. Our results also align with the overall CMIP5 and CMIP6 trends in the northward shift of precipitation anomalies north of SESA and the drying trend to the south of Brazil, although our pacemaker reveals a weakening of the dry anomalies driven by strong El Niño events over the Amazon basin. Future work will assess whether there is any saturation of dry anomalies over this area with El Niño events of large amplitude such as the ones we are focusing on.

In terms of future changes in surface temperature anomalies driven by strong El Niño, we find similarities between our results and the overall trend encompassing El Niño events of any amplitude. Some examples are a hot-gets-hotter trend over western Brazil, and Northern Africa, and while CMIP5 projections do not show a significant change over Australia^37^, our results agree with overall CMIP6 projections^38^ and large ensemble studies^36^.

Some additional features arise when focusing on strong El Niño events that are not seen in the overall projections of ENSO impacts based on composite analysis, that indicate either a nonlinear climate response to strong El Niño events or a disagreement of the EC-Earth3-CC model with the CMIP6 multi-mean results (i.e. McGregor et al.^38^). Our results reveal potential nonlinearities in the future climate response to El Niño events (e.g. the Amazon basin), where increasing SST anomalies in the Niño3.4 region do not further amplify the global surface impacts associated with a canonical El Niño event. This nonlinearities might be accompanied by shifts in circulation induced by climate change, with South America being the most illustrative example. According to our results, dry anomalies over the Amazon basin driven by very strong El Niño will get muted in the future, and wet and cool anomalies over the SESA are expected to shift northwards in DJF. Further work is needed to assess the nature of these shifts and nonlinear behaviours.

We find an intensification of cold anomalies driven by strong El Niño over northeast North America and a significant weakening of warm anomalies over northern South America and southern Africa. By comparing changes in the teleconnections of strong El Niño events with mean state changes due to climate change, we emphasise that although strong El Niño might drive cool anomalies in the future in some regions, the underlying signature of climate change largely dominates the global sign of temperature anomalies in the future, masking potential cooling effects of natural climate variability. The fact that changes in warm days driven by strong El Niño in the future over northern South America and Southern Africa are not very pronounced is caused by the use of fully moving thresholds (based on climatological values of each period) to compute these extreme heat indices. In the examples shown in this study, this indicates that thermodynamic changes modulated by climate change are the dominant source of the shift in the distribution of maximum daily temperature values, in agreement with Vogel et al.^56^. Future work is needed to address potential nonlinearities in the impacts driven by very strong El Niño events in the future.

As seen in most CMIP6 models, EC-Earth3-CC shows a long-term future trend resembling an El Niño-like warming in the tropical east Pacific, decreasing the zonal SST gradient by almost 20% towards the end of the 21^st^ century. This feature is consistent with a weakening Fof the Walker circulation and is similar to the trend projected in most CMIP5^57^ and CMIP6 models^27^, but does not match observed the Walker circulation strengthening and SST trends over recent decades^58,59^.

Human-caused climate change is warming the planet at unprecedented rates. Natural climate variability can either amplify or mute the signature of climate change in the future. Strong El Niño events are projected to increase in frequency^20^, and given the severity and reach of their impacts, it is crucial to understand how the impacts of these extreme events will change in the future to facilitate adaptation and mitigation policies in the areas affected. Our study suggests that the future climate conditions during strong El Niño events will be more impacted by the underlying signature of climate change than by changes in ENSO itself. Future studies are needed to improve our understanding of the mechanisms of such amplification and weakening of the future impacts of strong El Niño events. Additionally, a multi-model approach would enable our results to be verified for a larger set of projections, as well as an exploration of other seasons beyond the canonical boreal winter season.

## Methods

Pacemaker simulations are performed with the EC-Earth3-CC model^60^ to understand changes in the impacts of strong El Niño events between present and future climates.

### Model

EC-Earth3-CC is an Earth System model with an approximate atmosphere and ocean resolution of 1°. The atmospheric model is the European Center for Medium-range Weather Forecasts (ECMWF) Integrated Forecasting System (IFS) version 36r4, which is based on the ECMWF seasonal prediction system 4, and has a resolved stratosphere. NEMO3.6 and LIM3 are the ocean and sea ice components; biogeochemical processes in the ocean and over land are simulated by the PISCES and LPJ-GUESS models, respectively.

The EC-Earth3-CC model generally reproduces observed ENSO teleconnections. Figure S1 compares the global ENSO precipitation and surface temperature teleconnections in the December-February (DJF) season in EC-Earth3-CC with observations from the GPCP v2.3^61^ and Berkeley Earth^62^ datasets. For the model-data comparison we use output from a time-dependent tropical pacemaker simulation with EC-Earth3-CC covering the period 1900-2018, where historical SSTs are forced with observed SST anomalies in the tropical Pacific ocean. This ensures the ENSO events simulated in the model broadly follow those observed during the historical period. The model captures the sign and amplitude of ENSO-related precipitation anomalies over the tropical Pacific, but tends to overestimate the precipitation dipole response over the Indian Ocean (See Fig. S1). Over the equatorial Atlantic, the model displays a weak zonal precipitation dipole consisting of dry conditions over the Amazon and western equatorial Atlantic and wet anomalies over the eastern Atlantic and the Gulf of Guinea, whereas observations show an overall negative precipitation anomaly that peaks off the east Brazilian coast. Regardless of these differences, the overall patterns of global response in temperature and precipitation are well captured by EC-Earth3-CC.

### Experimental protocol

Six experiments are performed in which the model SSTs in the equatorial eastern Pacific are restored towards the observed SST anomalies from the 1982-1983, 1997-98 and 2015-16 strong El Niño events superimposed on either the present day or future model climatological SST. Observed monthly SST anomalies are extracted from the ERSSTv5 dataset^9^ calculated relative to the period 1981-2010. Present day climatological values are estimated from the historical simulations of EC-Earth3-CC using the period 2005-2014. For the future, we use the SSP2-4.5 scenario, a middle-of-the-road greenhouse gas emissions pathway where global mean surface temperature in EC-Earth3-CC increases by 2.8 K in 2100 relative to preindustrial times. This SSP is consistent with the climate that would be achieved by the end of this century if countries deliver on their current climate targets. Acknowledging that a stronger SSP scenario would provide a larger climate change signal, we performed large ensemble experiments with each El Niño event being simulated 30 times in both present and future climates, ensuring that changes in El Niño in the future are distinguishable from the climate change signal. The period 2085-2094 is used to define the future climatology. For each 10-year climatological period, we average over 10 initial condition ensemble members, meaning that the climatological states are calculated over 100 years.

The observed ERSSTv5 SST anomalies for the 3 strong El Niño cases are interpolated to the model’s ocean grid (ORCA1) and added to the present or future model climatology to produce the target restoring fields. For each experiment, a 30 member ensemble is generated by using 3 sets of initial conditions from each of the 10 historical or SSP2-4.5 simulations at 5 year intervals (for example for present-day we use 2005, 2010 and 2014). The SST target pattern is imposed through surface flux restoring with a restoring coefficient strong enough to achieve atmosphere-only conditions in the restoring domain, which spans across the tropical Pacific from latitudes is 10°S - 10°N and longitudes from 130°E - 70°W, with a 10° buffer zone on each side except the eastern margin which coincides with the American continent. All variables outside of the restoring region evolve freely in a coupled configuration. Each member is integrated for 2 years starting from January 1st and the SST restoring is activated from the 1st of January of the first year until the end of December of the second year, thus having a 2-year simulation for each ensemble member where SST anomalies are restored monthly to match the target observed pattern. The result is a suite of 30-member ensemble simulations for 3 strong El Niño events in present and future climates. The analysis showed very similar changes in impacts for the 3 El Niño cases, so the analysis focuses on the “multi-event mean”, hereafter MEM.

We focus on the DJF season when the El Niño events peak and compare the anomalous response to strong El Niño in the future with that of the present. Anomalies from each period are calculated relative to their corresponding climatological values.

### Climate signal decomposition

To differentiate between changes to El Niño itself and changes in El Niño impacts added on top of the global warming signal, the figures from this paper follow a similar structure. We first introduce the image showing present-day anomalies during very strong El Niño events in DJF (Eq. 1), consisting of subtracting the present-day climatology to the absolute climate impacts during El Niño:

1 $$ElNi\tilde{{\rm{n}}}oimpacts={ElNi\tilde{{\rm{n}}}o}_{p}-{Meanclimate}_{p}$$

We then explore changes in El Niño impacts in the future and present, removing the mean climate state and thus accounting just for changes to El Niño teleconnections regardless of the background climate change signal (Eq. 2):

2 $$Changes\in ElNi\tilde{{\rm{n}}}oimpacts=({ElNi\tilde{{\rm{n}}}o}_{f}-{Meanclimate}_{f})-({ElNi\tilde{{\rm{n}}}o}_{p}-{Meanclimate}_{p})$$

Changes in the mean climate state are also quantified and displayed in the plots (Eq. 3):

3 $$Meanstatechanges={Meanstate}_{f}-{Meanstate}_{p}$$

Adding up Eqs. 2 and 3 we obtain Eq. 4, which illustrate the changes in absolute impacts of El Niño in the future, considering both changes in El Niño teleconnections as well as changes in the mean state.

4 $$Totalchanges\in ElNi\tilde{{\rm{n}}}oimpacts={ElNi\tilde{{\rm{n}}}o}_{f}-{ElNi\tilde{{\rm{n}}}o}_{p}$$

### Climate indices

El Niño is known to drive weather extremes over North America^15,53^, northern South America and Southern Africa^2^, among other regions, so these are used as three regional case studies to examine the influence of future climate change on El Niño impacts.

In North America, we quantify the change in cool days per winter, which is computed as the number of days when maximum daily surface temperatures (Tmax) during the DJF season lay below the 10^th^ percentile based on the model climatology^63^. Secondly we explore changes in the amount of warm days per summer, which is again computed using maximum daily temperature and calculated the percentage change in days when maximum temperature exceeds the 90^th^ percentile. We focus on northern South America and Southern Africa to explore changes in the frequency of warm days per summer.

Note that the thresholds to compute cool and warm days in the present and future are based on the present (1995-2014) and future (2085-2094) climatologies, respectively, using fully moving thresholds^51^. Therefore, the indices capture similar relative anomalies in the two periods. While we do not link the meteorological conditions to climate impacts (e.g. on human health), this choice is most relevant to a situation in which society undertakes some climate adaptation to accommodate mean climate change. An alternative approach would be to use the present day threshold in both periods, which would be more relevant to a situation in which climate adaptation is not undertaken. To assess whether changes are statistically significant, we performed a two sided Student’s t-test.

## Supplementary information

Supplementary Information

## Data Availability

The code used to calculate the indicators and prepare the figures will be available upon request. The experimental protocol can be accessed in this Gitlab repository: https://earth.bsc.es/gitlab/ptrascas/el-nino-pacemaker-experimental-design.

## References

[1] Taschetto, A. S. et al. ENSO atmospheric teleconnections. *El Niño Southern Oscillation in a Changing Climate,* 309–335 (2020).

[2] Arblaster, J. M. & Alexander, L. V. The impact of the El Niño-Southern Oscillation on maximum temperature extremes. *Geophys. Res. Lett.*, **39**, (2012).

[3] Cai, W. et al. Climate impacts of the El Niño–Southern Oscillation on South America. *Nat. Rev. Earth Environ.* **1**, 215–231 (2020).

[4] Ratnam, J. V., Behera, S. K., Masumoto, Y. & Yamagata, T. Remote effects of El Niño and Modoki events on the austral summer precipitation of southern Africa. *J. Clim.* **27**, 3802–3815 (2014).

[5] Lieber, R., Brown, J., King, A. & Freund, M. Historical and Future Asymmetry of ENSO Teleconnections with Extremes. *J. Clim.* **37**, 5909–5924 (2024).

[6] Mooley, D. A. & Parthasarathy, B. Indian summer monsoon and El Niño. *Pure Appl. Geophysics* **121**, 339–352 (1983).

[7] Seager, R. et al. Mechanisms of ENSO-forcing of hemispherically symmetric precipitation variability. *Q. J. R. Meteorological Soc.: A J. Atmos. Sci., Appl. Meteorol. Phys. Oceanogr.* **131**, 1501–1527 (2005).

[8] Goddard, L. & Graham, N. E. Importance of the Indian Ocean for simulating rainfall anomalies over eastern and southern Africa. *J. Geophys. Res.: Atmospheres* **104**, 19099–19116 (1999).

[9] Huang, B. et al. Extended reconstructed sea surface temperature, version 5 (ERSSTv5): upgrades, validations, and intercomparisons. *J. Clim.* **30**, 8179–8205 (2017).

[10] Funk, C. et al. 18. Anthropogenic enhancement of moderate-to-strong El Niño events likely contributed to drought and poor harvests in southern Africa during 2016. *Bull. Am. Meteorol. Soc.* **99**, S91–S96 (2018).

[11] Panisset, J. S. et al. Contrasting patterns of the extreme drought episodes of 2005, 2010 and 2015 in the Amazon Basin. *Int. J. Climatol.* **38**, 1096–1104 (2018).

[12] Duke, N. C. et al. Large-scale dieback of mangroves in Australia’s Gulf of Carpentaria: a severe ecosystem response, coincidental with an unusually extreme weather event. *Mar. Freshw. Res.* **68**, 1816–1829 (2017).

[13] Page, S. E. et al. The amount of carbon released from peat and forest fires in Indonesia during 1997. *Nature* **420**, 61–65 (2002). 12422213 10.1038/nature01131

[14] Patra, P. K. et al. The Orbiting Carbon Observatory (OCO-2) tracks 2–3 peta-gram increase in carbon release to the atmosphere during the 2014–2016 El Niño*. Sci. Rep.* **7**, 13567 (2017). 10.1038/s41598-017-13459-0 PMC5648889 29051612

[15] Glantz, M. H. *Currents of change: impacts of El Niño and La Niña on climate and society*. (Cambridge University Press, 2001).

[16] Le Quéré, C. et al. Global carbon budget 2018. *Earth Syst. Sci. Data Discuss.* **2018**, 1–3 (2018).

[17] Wang, J. et al. Contrasting terrestrial carbon cycle responses to the 1997/98 and 2015/16 extreme El Niño events. *Earth Syst. Dyn.* **9**, 1–14 (2018).

[18] Antilla-Hughes, J. K., Jina, A. S. & McCord, G. C. ENSO impacts child undernutrition in the global tropics. *Nat. Commun.* **12**, 5785 (2021). 34642319 10.1038/s41467-021-26048-7 PMC8511020

[19] Santoso, A. et al. Late-twentieth-century emergence of the El Niño propagation asymmetry and future projections. *Nature* **504**, 126–130 (2013). 24240279 10.1038/nature12683

[20] Cai, W. et al. Increasing frequency of extreme El Niño events due to greenhouse warming. *Nat. Clim. Change* **4**, 111–116 (2014).

[21] Lengaigne, M. & Vecchi, G. A. Contrasting the termination of moderate and extreme El Niño events in coupled general circulation models. *Clim. Dyn.* **35**, 299–313 (2010).

[22] Chung, C. T., Power, S. B., Arblaster, J. M., Rashid, H. A. & Roff, G. L. Nonlinear precipitation response to El Niño and global warming in the Indo-Pacific. *Clim. Dyn.* **42**, 1837–1856 (2014).

[23] Taschetto, A. S. & England, M. H. El Niño modoki impacts on Australian rainfall. *J. Clim.* **22**, 3167–3174 (2009).

[24] Beniche, M. et al. A distinct and reproducible teleconnection pattern over North America during extreme El Niño events. *Sci. Rep.* **14**, 2457 (2024). 38291103 10.1038/s41598-024-52580-9 PMC10828491

[25] Salas, H. D. et al. Precipitation over northern South America and the far-eastern Pacific during ENSO: Phase synchronization at inter-annual time scales. *Int. J. Climatol.* **44**, 2106–2123 (2024).

[26] Trascasa-Castro, P., Maycock, A. C., Yiu, Y. Y. S. & Fletcher, J. K. On the linearity of the stratospheric and Euro-Atlantic sector response to ENSO. *J. Clim.* **32**, 6607–6626 (2019).

[27] Maher, N. et al. The future of the El Niño–Southern Oscillation: Using large ensembles to illuminate time-varying responses and inter-model differences. *Earth Syst. Dyn.* **14**, 413–431 (2023).

[28] Fredriksen, H. B., Berner, J., Subramanian, A. C. & Capotondi, A. How does El Niño–Southern Oscillation change under global warming—A first look at CMIP6. *Geophys. Res. Lett.* **47**, e2020GL090640 (2020).

[29] Cai, W. et al. Increased ENSO sea surface temperature variability under four IPCC emission scenarios. *Nat. Clim. Change* **12**, 228–231 (2022).

[30] Heede, U. K. & Fedorov, A. V. Colder eastern equatorial Pacific and stronger Walker circulation in the early 21^st^ century: separating the forced response to global warming from natural variability. *Geophys. Res. Lett.* **50**, e2022GL101020 (2023).

[31] Seager, R., Henderson, N. & Cane, M. Persistent discrepancies between observed and modeled trends in the tropical Pacific Ocean. *J. Clim.* **35**, 4571–4584 (2022).

[32] Lim, E. P. et al. Continuation of tropical Pacific Ocean temperature trend may weaken extreme El Niño and its linkage to the Southern Annular Mode. *Sci. Rep.* **9**, 17044 (2019). 31745225 10.1038/s41598-019-53371-3 PMC6864090

[33] Lee, J.-Y. et al. Future Global Climate: Scenario-Based Projections and Near-Term Information. In Climate Change 2021: The Physical Science Basis. Contribution of Working Group I to the Sixth Assessment Report of the Intergovernmental Panel on Climate Change [Masson-Delmotte, V., P. Zhai, A. Pirani, S.L. Connors, C. Péan, S. Berger, N. Caud, Y. Chen, L. Goldfarb, M.I. Gomis, M. Huang, K. Leitzell, E. Lonnoy, J.B.R. Matthews, T.K. Maycock, T. Waterfield, O. Yelekçi, R. Yu, and B. Zhou (eds.)]. *Cambridge University Press*, Cambridge, United Kingdom and New York, NY, USA, pp. 553–672, 10.1017/9781009157896.006 (2021).

[34] Hu, K., Huang, G., Huang, P., Kosaka, Y. & Xie, S. P. Intensification of El Niño-induced atmospheric anomalies under greenhouse warming. *Nat. Geosci.* **14**, 377–382 (2021).

[35] Ying, J. et al. Emergence of climate change in the tropical Pacific. *Nat. Clim. Change* **12**, 356–364 (2022).

[36] Fasullo, J. T., Otto-Bliesner, B. L. & Stevenson, S. ENSO’s changing influence on temperature, precipitation, and wildfire in a warming climate. *Geophys. Res. Lett.* **45**, 9216–9225 (2018).

[37] Perry, S. J., McGregor, S., Sen Gupta, A., England, M. H. & Maher, N. Projected late 21st century changes to the regional impacts of the El Niño-Southern Oscillation. *Clim. Dyn.* **54**, 395–412 (2020).

[38] McGregor, S., Cassou, C., Kosaka, Y. & Phillips, A. S. Projected ENSO teleconnection changes in CMIP6. *Geophys. Res. Lett.* **49**, e2021GL097511 (2022).

[39] Maher, N. et al. The future of the El Niño-Southern Oscillation: Using large ensembles to illuminate time-varying responses and inter-model differences. *Earth Syst. Dyn. Discuss.* **2022**, 1–28 (2022).

[40] Drouard, M. & Cassou, C. A modeling-and process-oriented study to investigate the projected change of ENSO-forced wintertime teleconnectivity in a warmer world. *J. Clim.* **32**, 8047–8068 (2019).

[41] Zhou, Z. Q., Xie, S. P., Zheng, X. T., Liu, Q. & Wang, H. Global warming–induced changes in El Niño teleconnections over the North Pacific and North America. *J. Clim.* **27**, 9050–9064 (2014).

[42] Cai, W. et al. Changing El Niño–Southern Oscillation in a warming climate. *Nat. Rev. Earth Environ.* **2**, 628–644 (2021).

[43] Dhage, L. & Widlansky, M. J. Assessment of 21st century changing sea surface temperature, rainfall, and sea surface height patterns in the tropical Pacific Islands using CMIP6 greenhouse warming projections. *Earth’s. Future* **10**, e2021EF002524 (2022).

[44] Brönnimann, S. Impact of El Niño–southern oscillation on European climate. *Rev. Geophys.* **45**, (2007).

[45] Firpo, M. Â. F. et al. Assessment of CMIP6 models’ performance in simulating present-day climate in Brazil. *Front. Clim.* **4**, 948499 (2022).

[46] Perkins-Kirkpatrick, S. E. & Gibson, P. B. Changes in regional heatwave characteristics as a function of increasing global temperature. *Sci. Rep.* **7**, 12256 (2017). 28947762 10.1038/s41598-017-12520-2 PMC5613001

[47] Adnan, M. S. G., Dewan, A., Botje, D., Shahid, S. & Hassan, Q. K. Vulnerability of Australia to heatwaves: A systematic review on influencing factors, impacts, and mitigation options. *Environ. Res.* **213**, 113703 (2022). 35716815 10.1016/j.envres.2022.113703

[48] Lee, S. K., Wang, C. & Mapes, B. E. A simple atmospheric model of the local and teleconnection responses to tropical heating anomalies. *J. Clim.* **22**, 272–284 (2009).

[49] Johnson, N. C., Wittenberg, A. T., Rosati, A. J., Delworth, T. L. & Cooke, W. Future changes in boreal winter ENSO teleconnections in a large ensemble of high-resolution climate simulations. *Front. Clim.* **4**, 941055 (2022).

[50] Geng, X., Kug, J. S. & Kosaka, Y. Future changes in the wintertime ENSO-NAO teleconnection under greenhouse warming. *npj Clim. Atmos. Sci.* **7**, 81 (2024).

[51] Beverley, J. D., Collins, M., Lambert, F. H. & Chadwick, R. Future changes to El Niño teleconnections over the north Pacific and North America. *J. Clim.* **34**, 6191–6205 (2021).

[52] Kim, J. S., Kug, J. S. & Jeong, S. J. Intensification of terrestrial carbon cycle related to El Niño–Southern Oscillation under greenhouse warming. *Nat. Commun.* **8**, 1674 (2017). 29162846 10.1038/s41467-017-01831-7 PMC5698330

[53] Meehl, G. A. & Teng, H. Multi-model changes in El Niño teleconnections over North America in a future warmer climate. *Clim. Dyn.* **29**, 779–790 (2007).

[54] Beverley, J. D., Collins, M., Lambert, F. H. & Chadwick, R. Drivers of changes to the ENSO–Europe teleconnection under future warming. *Geophys. Res. Lett.* **51**, e2023GL107957 (2024).

[55] Russo, S., Marchese, A. F., Sillmann, J. & Immé, G. When will unusual heat waves become normal in a warming Africa? *Environ. Res. Lett.* **11**, 054016 (2016).

[56] Vogel, M. M., Zscheischler, J., Fischer, E. M. & Seneviratne, S. I. Development of future heatwaves for different hazard thresholds. *J. Geophys. Res.: Atmospheres* **125**, e2019JD032070 (2020). 10.1029/2019JD032070 PMC7380308 32728502

[57] Cai, W. et al. ENSO and greenhouse warming. *Nat. Clim. Change* **5**, 849–859 (2015).

[58] Kociuba, G. & Power, S. B. Inability of CMIP5 models to simulate recent strengthening of the Walker circulation: Implications for projections. *J. Clim.* **28**, 20–35 (2015).

[59] Heede, U. K. & Fedorov, A. V. Eastern equatorial Pacific warming delayed by aerosols and thermostat response to CO_2_ increase. *Nat. Clim. Change* **11**, 696–703 (2021).

[60] Döscher, R. et al. The EC-earth3 Earth system model for the climate model intercomparison project 6. *Geoscientific Model Dev. Discuss.* **2021**, 1–90 (2021).

[61] Adler, R. et al. The new version 2.3 of the Global Precipitation Climatology Project (GPCP) monthly analysis product. *University of Maryland, April*, 1072-1084, (2016).

[62] Rohde, R. A. & Hausfather, Z. The Berkeley Earth land/ocean temperature record. *Earth Syst. Sci. Data* **12**, 3469–3479 (2020).

[63] Donat, M. G. et al. Updated analyses of temperature and precipitation extreme indices since the beginning of the twentieth century: The HadEX2 dataset. *J. Geophys. Res.: Atmospheres* **118**, 2098–2118 (2013).

