# Supplementary material for: Future climate response to observed strong El Niño analogues
Source: NPJ Clim Atmos Sci. 2025 Mar 21;8(1):116. doi: 10.1038/s41612-025-01003-1 (PMC11928316; doi:10.1038/s41612-025-01003-1)
Supplement: Supplementary file 1 — Supplementary Information [file 41612_2025_1003_MOESM1_ESM.pdf]

Future climate response to observed strong El Niño analogues

Paloma Trascasa-Castro[1,2], Yohan Ruprich-Robert[1] and Amanda C. Maycock[2]

[1] Earth Sciences Department, Barcelona Supercomputing Center, Barcelona, Spain.
[2] School of Earth and Environment, University of Leeds, Leeds, United Kingdom.

Supporting information

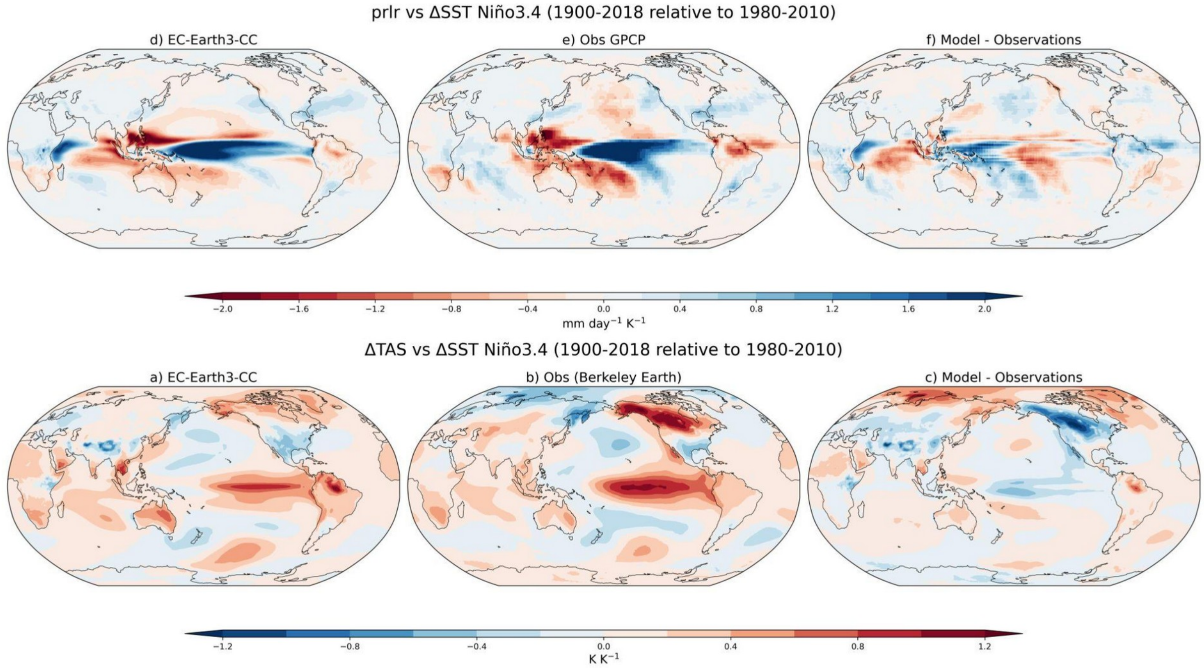

Figure S1. EC-Earth3-CC mean precipitation (top) and surface temperature (bottom) anomalies regressed onto SST anomalies in the Niño3.4 region in DJF (left column), compared to observations (middle column) and the difference between both. Model data corresponds to output of a transient pacemaker simulation run with the EC-Earth3-CC model where observed SST anomalies in the tropical Pacific are imposed on top of the model's own climatology between 1900 and 2018. SST and TAS anomalies are computed relative to the 1980-2010 period, and the Berkeley Earth dataset (Rohde et al. 2020) is used as the reference observational dataset. For precipitation, the period used spans from 1980 to 2018, and GPCP v2.3 (Adler et al. 2016) is used as the observational dataset of reference. We stress that the sample size of the observed ENSO composites shown in the supplement is larger for temperature (120 years) than for precipitation (40 years).

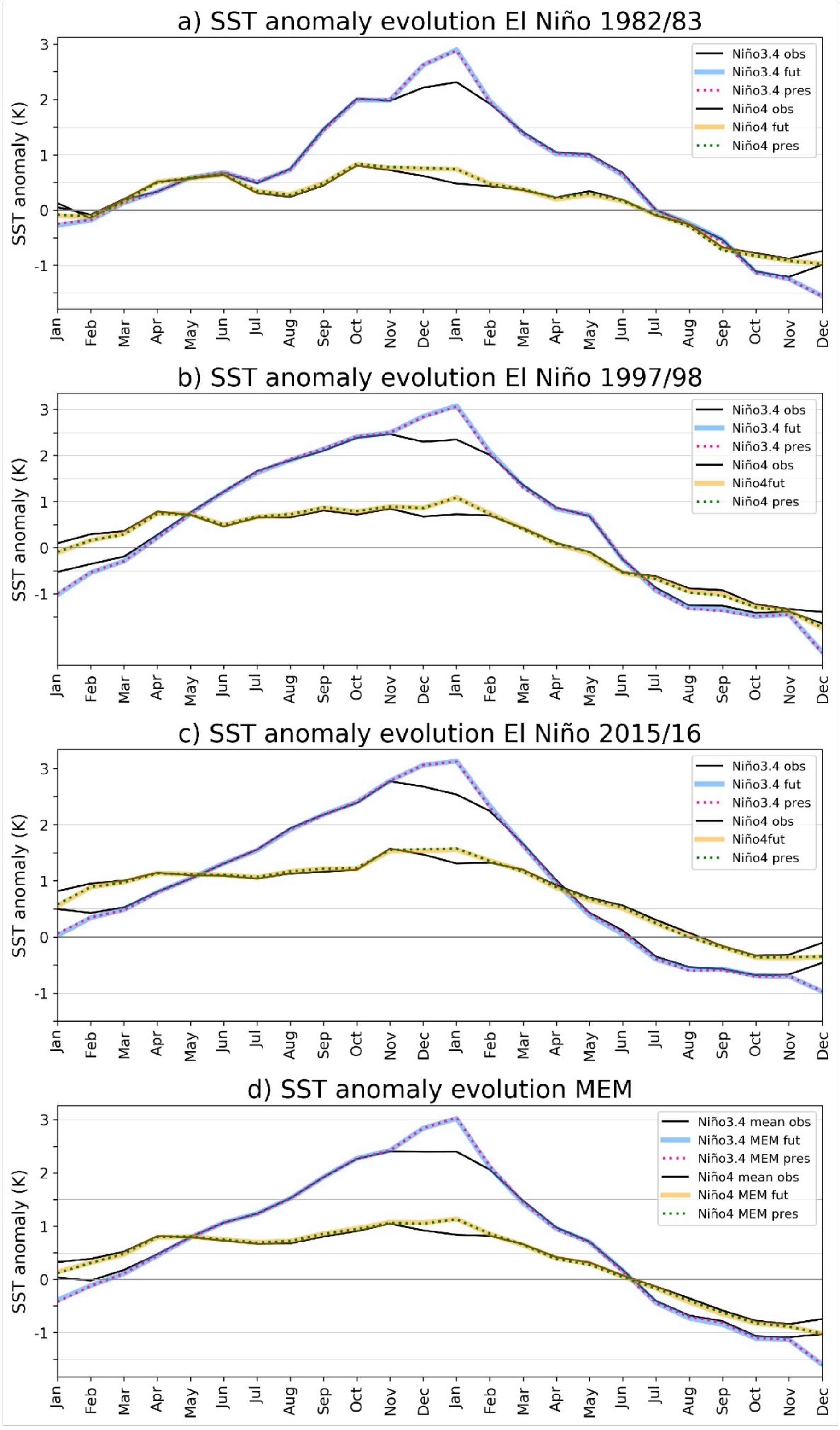

Figure S2. SST evolution during the 3 strong El Niño events (a) 1982/83, b) 1997/98, c) 2015/16) and d) multi event mean.Solid black lines correspond to SST anomalies from observations (ERSSTv5). Dashed lines correspond to present-day SST anomalies in the Niño3.4 (red) and Niño4 (black) regions in our pacemaker simulations. Thick lines correspond to SST anomalies in the future simulation in the Niño3.4 (blue) and Niño4 (yellow) regions.

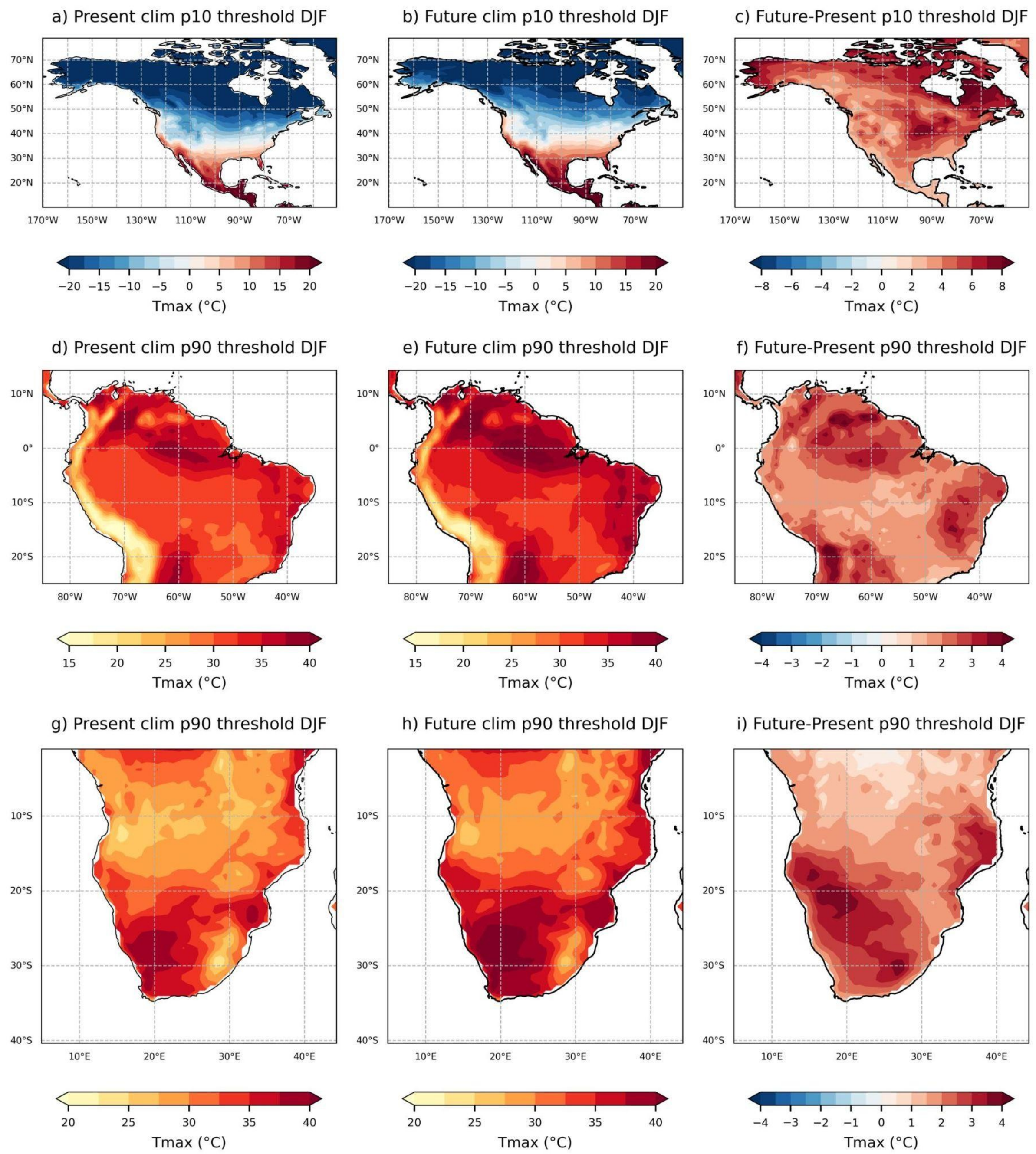

Figure S3. Climatological thresholds for cool (a-c) and warm (d-i) days over North America (first row), Australia (middle row) and Southern Africa (bottom row) in the present day (first column) and future climatological states (middle column), as well as the difference between future and present day thresholds (third column) in the EC-Earth3-CC model. Present day climatologies cover the period from 2005 to 2014 and the future climatologies span from 2085 to 2094 following the SSP2-4.5.
